# Sympathetic Nervous System Activation and Its Modulation: Role in Atrial Fibrillation

**DOI:** 10.3389/fnins.2018.01058

**Published:** 2019-01-23

**Authors:** Revathy Carnagarin, Marcio G. Kiuchi, Jan K. Ho, Vance B. Matthews, Markus P. Schlaich

**Affiliations:** ^1^Dobney Hypertension Centre, School of Medicine, Royal Perth Hospital Unit, Medical Research Foundation, The University of Western Australia, Perth, WA, Australia; ^2^Departments of Cardiology and Nephrology, Royal Perth Hospital, Perth, WA, Australia; ^3^Neurovascular Hypertension and Kidney Disease Laboratory, Baker Heart and Diabetes Institute, Melbourne, VIC, Australia

**Keywords:** autonomic nervous system, hypertension, neuromodulation, atrial fibrillation, sympathetic overdrive

## Abstract

The autonomic nervous system (ANS) has a significant influence on the structural integrity and electrical conductivity of the atria. Aberrant activation of the sympathetic nervous system can induce heterogeneous changes with arrhythmogenic potential which can result in atrial tachycardia, atrial tachyarrhythmias and atrial fibrillation (AF). Methods to modulate autonomic activity primarily through reduction of sympathetic outflow reduce the incidence of spontaneous or induced atrial arrhythmias in animal models and humans, suggestive of the potential application of such strategies in the management of AF. In this review we focus on the relationship between the ANS, sympathetic overdrive and the pathophysiology of AF, and the potential of sympathetic neuromodulation in the management of AF. We conclude that sympathetic activity plays an important role in the initiation and maintenance of AF, and modulating ANS function is an important therapeutic approach to improve the management of AF in selected categories of patients. Potential therapeutic applications include pharmacological inhibition with central and peripheral sympatholytic agents and various device based approaches. While the role of the sympathetic nervous system has long been recognized, new developments in science and technology in this field promise exciting prospects for the future.

## Cardiac Autonomic Nervous System (ANS) Anatomy

The heart is extensively innervated and effectively regulated by the autonomic nervous system (ANS) through its sympathetic and parasympathetic branches (Kimura et al., 2012). The cardiac neural control occurs at multiple levels with each level capable of parallel processing of afferent neurotransmission and efferent cardiac sympathetic outflow (Esler, 2004). The ganglion cells of the ANS are located either inside (intrinsic) or outside the heart (extrinsic) and play an important role in cardiac function and arrhythmogenesis. The human cardiac intrinsic nervous system is made of ganglionated plexi (GP), which contain local circuit neurons of many types and chemo- and mechanosensory neurons that are distributed across the heart (Schotten et al., 2011). The GP are typically well innervated with both adrenergic and vagal nerve terminals and are accommodated in the fat pads, which are located mainly around the pulmonary vein ostia. The extrinsic sympathetic innervation is mediated via the cervical, stellate (cervicothoracic), and thoracic ganglia. Parasympathetic extrinsic innervation is transmitted via the vagus nerve, although sympathetic fibers are located in vagal nerves and parasympathetic fibers in sympathetic nerves as well (Kawashima, 2005; Seki et al., 2014).

**FIGURE 1:**
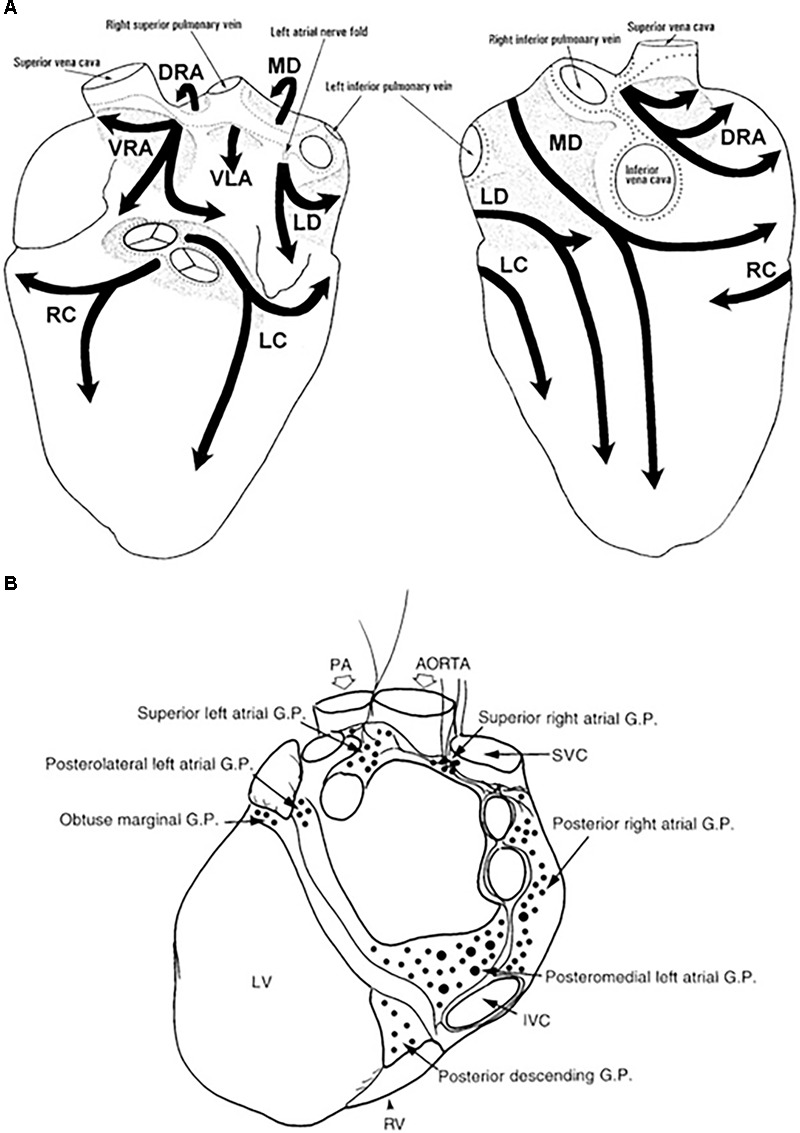
Anatomy of the intrinsic autonomic nervous system. The extensive network of epicardial nerves on the atria and ventricles are divided in 7 subplexi: **(A)** Left is a posterior and right is an anterior view of the heart. Along these subplexi, ganglia are localized, conglomerated in ganglion plexi (GP) marked in light gray (Pauza et al., 2000). **(B)** The major atrial and ventricular GP from a posterior view of the heart (Armour et al., 1997). DRA, dorsal right atrial ganglionated subplexus; IVC, inferior vena cava; LC, left coronary ganglionated subplexus; LD, left dorsal ganglionated subplexus; LV, left ventricle; MD, middle dorsal ganglionated subplexus; PA, pulmonary artery; RC, right coronary ganglionated subplexus; RV, right ventricle; SVC, superior vena cava; VLA, ventral left atrial ganglionated subplexus; VRA, ventral right atrial ganglionated subplexus.

The extrinsic nerves run through the hilum of the heart along the great cardiac vessels and divide into seven epicardial subplexi, the intrinsic neural pathways of the ANS (Pauza et al., 2000). Small nerve fibers create a vast neural complex of small interconnecting efferent and afferent sympathetic, parasympathetic, and mixed nerve fibers, that contain the neurotransmitters, such as noradrenaline and acetylcholine, respectively, but some also include neuropeptide Y, somatostatin, vasoactive intestinal polypeptide, and substance P (Marron et al., 1994, 1995; Armour et al., 1997; Esler et al., 2006a; Tan et al., 2006; Deneke et al., 2011). The density of small fibers and ganglia is most significant in the posterior zone of the left atrium and surrounding the antrum of the left pulmonary veins (PVs) (Chevalier et al., 2005; Tan et al., 2006). The atria are mostly parasympathetically innervated, whereas the ventricles are primarily innervated by sympathetic nerve fibers (where only 16% of total cardiac ganglia reside) (Pauza et al., 2000; Kawano et al., 2003; Petraitiene et al., 2014). GP are clusters of ganglia from different subplexi and function as an intersection point of parasympathetic and sympathetic nerves and interconnect the intrinsic ANS (Armour et al., 1997; Hou et al., 2007; Malcolme-Lawes et al., 2013). The atrial GP are placed adjacent to the sinus node and PVs and are present in epicardial fat pads (Figure 1). Ventricular GP are located near the interventricular groove (Armour et al., 1997). The ligament of Marshall, the embryonic remnant of the left superior caval vein, close to the left superior PV is extensively innervated with parasympathetic and sympathetic nerves (Kim et al., 2000; Ulphani et al., 2007).

## Pathophysiology of Atrial Fibrillation

Autonomic nervous system activation has been well-known as a central determining factor of atrial arrhythmogenesis, (Sharifov et al., 2004; Chen and Tan, 2007; Shen et al., 2011; Shen and Zipes, 2014). Studies involving modulation of the sympathetic limb of ANS demonstrated that suppression of sympathetic tone leads to a notable reduction in atrial vulnerability to AF induction and post-ablation AF recurrence (Linz et al., 2012; Pokushalov et al., 2012). The prevalence of AF increases with age, affecting around 0.5% of the general population under 40 years of age, >5% of the general population over 65 years of age, and >10% of the general population over 80 years of age (Sankaranarayanan et al., 2013). In young patients, there was a shift toward vagal dominance in lone AF and nocturnal paroxysmal AF predominantly (Jayachandran et al., 2000). In patients with paroxysmal AF and syncope, there is an abnormal neural response even during sinus rhythm, at which AF triggers vasovagal syncope (Brignole et al., 1993). It is also important to highlight that AF in younger patients is associated with higher mortality rate than matched controls. Patients admitted into the hospital with incidental AF had a worse prognosis when compared to patients without AF, with higher risk of all-cause mortality in the younger age group when compared to the over-75-year-old population (Andersson et al., 2013).

Atrial fibrillation promotes shortening of the atrial refractory period and AF cycle length during the first days of the arrhythmia, mostly due to downregulation of the Ca^2+^-inward current and upregulation of inward rectifier K^+^ currents (Van Wagoner et al., 1999; Dobrev et al., 2005). Conversely, the structural cardiac disease leads to an extension of the atrial refractory period, demonstrating the heterogeneity of mechanisms causing AF in different patients (Schotten et al., 2011). Hyper-phosphorylation of various Ca^2+^-handling proteins may contribute to increasing spontaneous Ca^2+^ release events and eliciting activity (Voigt et al., 2012, 2014), thus provoking atrial ectopic beats, and thereby AF. Interestingly, the instability theory of Ca^2+^-handling has been recently challenged (Christ et al., 2014; Greiser et al., 2014), raising the hypothesis that Ca^2+^-induced calcium release may mediate AF in structurally remodeled atria, demonstrating how a modified autonomic tone can cause AF (Nguyen et al., 2009).

### Focal Initiation and Maintenance of AF

Haissaguerre et al. (2014) reported that a focal source in the pulmonary veins could initiate AF, and ablation of this source could eliminate recurring AF (Haissaguerre et al., 1998). The mechanisms of focal activity perhaps cover both triggered activity and local re-entry (Atienza et al., 2006; Patterson et al., 2007). Hierarchical organization of AF with rapidly activated zones promoting the arrhythmia has been demonstrated in individuals with paroxysmal AF (Mandapati et al., 2000; Sahadevan et al., 2004). However, it seems that the organization of these rapidly activated zones are less well demarcated in patients with persistent AF (Sanders et al., 2006).

### The Multiple Wavelet Hypothesis and Rotors as Sources of AF

The multiple wavelets and organized sources theories, though unclear are the two principal proposed paradigms of AF perpetuation. Moe et al. (1964) proposed, through a mathematical model, that multiple wavelets randomly propagating through the atria are capable of perpetuating AF which in turn provoke wave breaks, and generates new daughter wavelets. Though AF would be sustained as long as the number of wavelets is beyond a critical level, it is still unknown whether these wavelets are driving AF or if they are simply passive, and result from the breakup of more organized waves. Whilst the multiple wavelet concept is supported by theoretical rationale (Moe and Abildskov, 1959), experimental and clinical data (Cox et al., 1991), optical mapping techniques identified that wavelets resulting from the breakup of high frequency organized waves were not capable of maintaining AF independently (Chen et al., 2000). In patients with longstanding AF, the fibrillatory waves are due to epicardial breakthrough of waves that propagate in the deep layers of the atrial wall providing a constant independent source originating over the entire epicardial surface (Allessie et al., 2010; de Groot et al., 2010). Early ablative termination of AF in the PV may not completely abolish the presence of PV antrum wavelets over time, which can in turn cause AF (Haissaguerre et al., 2005; Calkins et al., 2012) resulting in limited clinical impact (Beukema et al., 2008).

Studies showed the presence of high-frequency spiral wave-like activity emanating regularly from the left atrium, subsequently driving irregular fibrillatory conduction in the rest of the atria (Schuessler et al., 1992; Mandapati et al., 2000; Verheule et al., 2010) whilst complex signal morphologies were associated with areas of slow conduction, wave collision, fibrillatory conduction, turning into wavelet pivot points and rotor meandering, or autonomic activation (Lu et al., 2008; Vaquero et al., 2008; Zlochiver et al., 2008). Localized source premise is based on experimental models in which organized re-entrant circuits, called “rotors” (Skanes et al., 1998; Vaquero et al., 2008) or focal impulses (Sahadevan et al., 2004) disorganize into AF. Jalife and co-workers clearly demonstrated that pacing-induced AF was due to the presence of a very rapid rotor (15–20 Hz) in the left atrium (Jalife et al., 2002). The CONFIRM trial demonstrated for the first time that localized sources in the form of electrical rotors and focal impulses are capable of sustaining AF in humans (Narayan et al., 2012). Furthermore, they also proved that brief ablation (Focal Impulse and Rotor Modulation, FIRM) of patient-specific AF-sustaining sources was able to terminate or consistently reduce persistent or paroxysmal AF prior to any conventional ablation in 86% of patients, and substantially increase long-term AF elimination compared to traditional AF ablation alone (Narayan et al., 2012). However, the results of the CONFIRM trial were limited to small series of patients with mixed AF types, so the actual role of stable rotors in more persistent forms remains to be proven. Further, in the absence of convincing evidence that human AF is driven by a single rotor, it is puzzling how we should interpret the CONFIRM study. In itself, this observation does not prove that human AF is driven by a single rapid source and the reported clinical success needs to be confirmed by other centers, especially in patients with longstanding persistent AF (Allessie and de Groot, 2014). Moreover, these studies utilize a low-resolution mapping system, which is not completely satisfactory in delivering mechanistic insights to draw conclusions (Ravelli and Mase, 2014). However, the high success rate of AF termination by targeting identified local sources strongly supports the hypothesis of rapidly organizing sources as key for the maintenance of AF.

## Clinical Conditions that may Act as Predisposing Factors to AF

**FIGURE 2:**
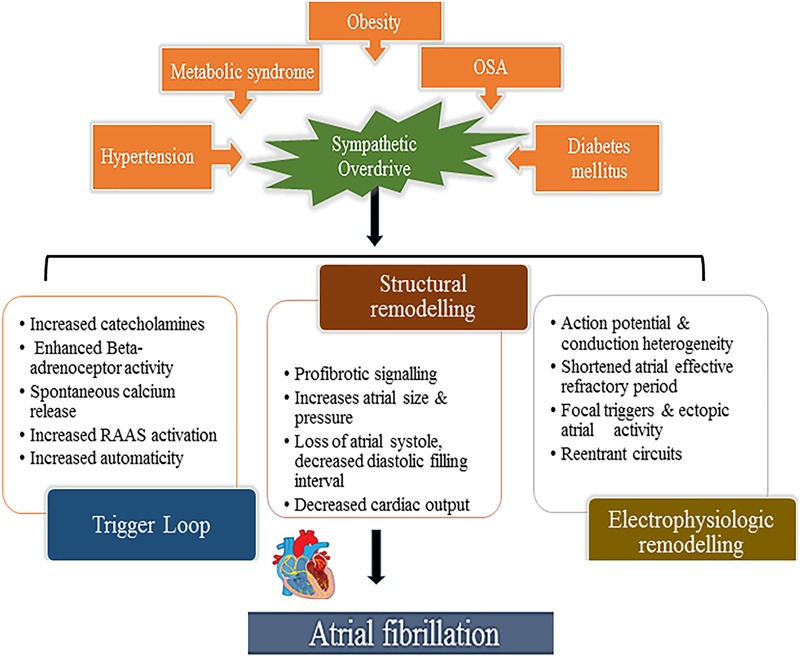
Aberrant sympathetic activation in conditions such as obesity (and particularly increased epicardial fat), hypertension, obstructive sleep apnea, diabetes mellitus and metabolic syndrome plays a fundamental role in the development of AF. Sympathetic activation through excess catecholamines in the circulation which increases calcium entry and the spontaneous release from the myocardial sarcoplasmic reticulum leading to enhanced automaticity (trigger loop) and enhanced renin-angiotensin-aldosterone provokes pro-fibrotic signaling in the myocardium altering atrial size, pressure and consequent structural remodeling thereby inducing focal triggers and atrial ectopies. This occurs hand in hand with atrial electrical remodeling with shortened action potential duration, conduction disturbances, also facilitating re-entry circuits, promoting and sustaining AF.

Multiple cardiovascular diseases and associated conditions increase the risk of developing AF (Table 1), recurrent AF, and AF-related consequences. Diagnosing such conditions, as well as preventing and treating them are vital to prevent AF and its disease burden. Knowledge of these factors and their management is therefore important for optimal treatment of AF patients (Abed et al., 2013; Pathak et al., 2014). The ANS exerts significant control on both cardiac electrophysiology as well as conditions such as, hypertension which is often associated with exaggerated sympathetic tone and is the single most important clinical factor that accounts for around 80% of AF (Schotten et al., 2011). This is further complicated by the associated atrial remodeling and dilatation that increases the probability of repetitive firing or even the presence of episodic re-entrant activation circuits. Other clinical conditions commonly associated with AF include cardiomyopathy, valvular and coronary heart disease, heart failure, metabolic syndrome and diabetes, suggested to contribute to around 20–30% of AF cases (Psaty et al., 1997; Schoonderwoerd et al., 2008; Mahfoud et al., 2011; Schotten et al., 2011; Selmer et al., 2012; Schnabel et al., 2015; Vermond et al., 2015). These clinical pathologies induce electrical, structural and autonomic atrial remodeling which results in conduction abnormalities such as rapidly firing focus and multiple complex (Buch et al., 2003; Gami et al., 2007; Aizer et al., 2009; Chamberlain et al., 2011; Kim et al., 2014; Larsson et al., 2014) re-entrant circuits, thereby AF (Figure 2). In addition, increased renin-angiotensin-aldosterone system activity associated with sympathetic overdrive induced profibrotic cardiac signaling, atrial fibrosis and fibrillation (Kumagai et al., 2003; Ehrlich et al., 2006; Baber et al., 2011; Reil et al., 2012).

**Table 1:** Cardiovascular and other conditions independently associated with atrial fibrillation.

Characteristic/comorbidity	Association with AF
Genetic predisposition (based on multiple common gene variants associated with AF), ccc specified in Table 1.	HR range 0.4–3.2
Older age	HR:
50–59 years	1.00 (reference)
60–69 years	4.98 (95% CI 3.49–7.10)
70–79 years	7.35 (95% CI 5.28–10.2)
80–89 years	9.33 (95% CI 6.68–13.0)
Hypertension (treated) vs. none	HR 1.32 (95% CI 1.08–1.60)
Heart failure vs. none	HR 1.43 (95% CI 0.85–2.40)
Valvular heart disease vs. none	RR 2.42 (95% CI 1.62–3.60)
Myocardial infarction vs. none	HR 1.46 (95% CI 1.07–1.98)
Thyroid dysfunction	(reference: euthyroid)
Hypothyroidism	HR 1.23 (95% CI 0.77–1.97)
Subclinical hyperthyroidism	RR 1.31 (95% CI 1.19–1.44)
Overt hyperthyroidism	RR 1.42 (95% CI 1.22–1.63)
Obesity	HR:
None (BMI < 25 kg/m^2^)	1.00 (reference)
Overweight (BMI 25–30 kg/m^2^)	1.13 (95% CI 0.87–1.46)
Obese (BMI ≥ 31 kg/m^2^)	1.37 (95% CI 1.05–1.78)
Diabetes mellitus vs. none	HR 1.25 (95% CI 0.98–1.60)
Chronic obstructive pulmonary disease	RR:
FEV1 ≥ 80%	1.00 (reference)
FEV1 60–80%	1.28 (95% CI 0.79–2.06)
FEV1 < 60%	2.53 (95% CI 1.45–4.42)
Obstructive sleep apnoea vs. none	HR 2.18 (95% CI 1.34–3.54)
Chronic kidney disease	OR:
None	1.00 (reference)
Stage 1 or 2	2.67 (95% CI 2.04–3.48)
Stage 3	1.68 (95% CI 1.26–2.24)
Stage 4 or 5	3.52 (95% CI 1.73–7.15)
Smoking	HR:
Never	1.00 (reference)
Former	1.32 (95% CI 1.10–1.57)
Current	2.05 (95% CI 1.71–2.47)
Alcohol consumption	RR:
None	1.00 (reference)
1– 6 drinks/week	1.01 (95% CI 0.94–1.09)
7–14 drinks/week	1.07 (95% CI 0.98–1.17)
15–21 drinks/week	1.14 (95% CI 1.01–1.28)
>21 drinks/week	1.39 (95% CI 1.22–1.58)
Habitual vigorous exercise	RR:
Non-exercisers	1.00 (reference)
<1 day/week	0.90 (95% CI 0.68-1.20)
1-2 days/week	1.09 (95% CI 0.95-1.26)
3-4 days/week	1.04 (95% CI 0.91-1.19)
5-7 days/week	1.20 (95% CI 1.02-1.41)

*AF, atrial fibrillation; BMI, body mass index; CI, confidence interval; FEV1, forced expiratory volume in 1 s; HR, hazard ratio; OR, odds ratio; RR, risk ratio. Adapted from Kirchhof et al. (2016).*

Autonomic remodeling may modify the reaction to ANS stimulation and the balance between the parasympathetic and sympathetic innervation (Coumel, 1996; Patterson et al., 2005; Furukawa et al., 2009; Ng et al., 2011; Shen et al., 2011). Increased sympathetic nerve density has been described in patients with permanent AF, but whether this is compensatory to increased vagal stimulation or causative of AF is unknown (Gould et al., 2006; Deneke et al., 2011). A heterogeneous increase in sympathetic innervation in the atria of dogs on rapid atrial pacing for long periods increases AF susceptibility (Jayachandran et al., 2000). Increased sympathetic and vagal nerve discharges before the onset of atrial arrhythmias in dogs with pacing-induced congestive heart failure by direct nerve recordings from the stellate ganglia and vagal nerves was also reported (Ogawa et al., 2007). Undeniably, atrial tachyarrhythmias in dogs presenting with congestive heart failure by fast cardiac pacing were prevented by prophylactic ablation of the stellate ganglion and the T2 to T4 thoracic sympathetic ganglia (Ogawa et al., 2007). In the same animal model of heart failure, Ng et al., also demonstrated increased sympathetic and parasympathetic nerve growth mostly in the pulmonary veins and the posterior wall of the left atrium (Ng et al., 2011).

In humans, PV ectopy, often originating from superior PVs, normally triggers paroxysmal AF (Haissaguerre et al., 1998). Animal and human studies have shown that stimulation of the GP close to PV triggers PV ectopies (Scherlag et al., 2005; Lim et al., 2011). In canine PVs, parasympathetic activation decreased action potential duration, while sympathetic activation increased myocardial cytoplasmatic (Ca^2+^) (Patterson et al., 2005). Both components were necessary for early afterdepolarizations in PVs, consequently triggering AF. Ectopic activity in other highly innervated structures (ligament of Marshall) possibly also triggers AF (Chevalier et al., 2005; Scherlag et al., 2005; Tan et al., 2006). During catheter ablation procedures stimulation of the ligament of Marshall caused ectopic beats and triggered AF (Báez-Escudero et al., 2014). On the other hand, AF triggers are not restrained to densely innervated tissue alone. A study reported that in ∼30% of patients who required a repeat PV isolation ectopic firing from the left atrial appendage was detected (Di Biase et al., 2010).

Intense atrial fibrosis formation and cardiomyocyte hypertrophy are common features of structural remodeling. Atrial tissue fibrosis damages electrophysiological cell-to-cell coupling and conduction (Allessie et al., 2001; Nattel, 2002; Iwasaki et al., 2011; Schotten et al., 2011; Wakili et al., 2011). Long-term AF induces myocyte hypertrophy and increases endomysial fibrosis. This is accompanied by dissociated conduction and electrical dissociation between the epicardial layer and the endocardial bundle connections frequently promoting permanent forms of AF (Eckstein et al., 2011; Schotten et al., 2011). Conditions other than AF can also lead to atrial structural remodeling including chronic arterial hypertension which has been shown to cause electro-structural changes characterized by conduction abnormalities, atrial inflammation and fibrosis thereby increasing the risk of AF induction (Lau et al., 2010). Congestive heart failure induces structural remodeling characterized by increased fibrosis and changes in gap junctions, provoking conduction heterogeneity which promotes formation of micro-re-entry and macro-re-entry circuit pathways (Li et al., 1999). Long-term obstructive sleep apnoea is linked to significant atrial remodeling characterized by atrial enlargement, site-specific and extensive conduction abnormalities, as well as delayed sinus node recovery time in humans (Witkowski et al., 2011; Dimitri et al., 2012). The described alterations may have a crucial role in the generation of AF substrates.

Sympathetic activation also plays a major role in atrial arrhythmias following cardiac surgeries and post-operative (post-op) AF is associated with reduced long term survival with cardiac surgeries such as the coronary bypass and valvular surgeries especially the aortic valve replacement (Girerd et al., 2011). Often finding suggestive of enhanced sympathetic activation such as elevated nor-epinephrine levels (Kalman et al., 1995), increased sinus rate, atrial ectopy and time- and frequency-domain parameters of heart rate variability are associated with the onset of AF following cardiac surgeries (Dimmer et al., 1998; Amar et al., 2003). Ventral cardiac denervation significantly reduced the incidence and severity of AF in patients undergoing low-risk coronary bypass surgery (Melo et al., 2004). Post-operative AF is also common in patients who have undergone lung transplantation rather than cardiac transplantation, an effect attenuated by cardiac autonomic denervation that occurs in heart transplant patients (Dizon et al., 2009). Moreover, in a large series of patients following orthotopic cardiac transplant, macro re-entrant tachycardias (flutter and scar re-entry) seem to be the most common supraventricular arrhythmias in stable patients which are often supressed by catheter ablation. The blockade of β-receptors in patients presenting with acute post-operative AF has been effective in preventing recurrence of this arrhythmia after successful cardioversion (Kühlkamp et al., 2000; Nergårdh et al., 2007). Though post-op AF is of multifactorial etiology, sympathetic activation appears to be the most relevant predisposing mechanism of post-op AF and in line with this, the 2010 European Society of Cardiology guidelines recommend β-blocker treatment as first-line therapy in preventing post-op AF following cardiac surgeries (Camm et al., 2010).

## Sympatholytic Therapy – a Possible Strategy in the Management of AF?

### Pharmacological Sympathetic Inhibition

Given the contribution of sympathetic overdrive in the development of AF, sympathetic inhibition represents a logical therapeutic approach in the management of AF and the frequently coexisting hypertension, thereby alleviating the associated cardiovascular mortality and morbidity. Autonomic dysfunction characterized by sympathetic overdrive in scenarios of organic heart disease exhibits both electrical and structural remodeling of atrial myocardium in AF. Pharmacological sympatholysis can be achieved by using drugs such as clonidine and moxonidine. Clonidine is a centrally acting α2-adrenergic agonist that supresses the sympathetic outflow in the lower brainstem (Flacke et al., 1987), the preganglionic splanchnic nerve fibers and in postganglionic cardiac nerves fibers (Langer et al., 1980). In addition, clonidine prolongs atrioventricular node refractoriness by stimulation of parasympathetic outflow with beneficial effects on the control of ventricular response in patients with new-onset rapid AF (Roth et al., 1992). Moxonidine is an imidazoline I1-receptor agonist that inhibits sympathetic outflow at the level of the rostral ventrolateral medulla. In addition to effective blood pressure (BP) lowering and metabolic benefits (Haenni and Lithell, 1999; Chazova et al., 2006), moxonidine has been demonstrated to suppress atrial arrhythmogenesis (Lepran and Papp, 1994; Edwards et al., 2012; Cagnoni et al., 2016). In a subsequent study, moxonidine significantly increased the threshold dose of ouabain-induced cardiac arrhythmia by decreasing sympathetic tone (Mest et al., 1995). Moreover, moxonidine induced sympathetic inhibition decreased the AF burden in hypertensive patients with paroxysmal AF without any side effects (Deftereos et al., 2013). Furthermore, moxonidine reduced post-ablation recurrence of AF in hypertensive patients who underwent pulmonary vein isolation for drug-refractory paroxysmal AF (Giannopoulos et al., 2014).

Moxonidine seems specifically useful in obesity related hypertension where the sympathetic overdrive sustains both obesity and BP elevation. With the well-established relationship between abdominal obesity, the metabolic syndrome, and the development of AF, obese hypertensives are at a greater risk for AF. Studies have identified that autonomic dysfunction occurs early in the pathophysiology of AF and precedes the development of metabolic abnormalities such as insulin-resistance and obesity (Huggett et al., 2006; Julius and Jamerson, 1994; Palatini et al., 1999) which in turn maintain sympathetic activation via release of adipokines and other bioactive cytokines from visceral fat depots (Grassi et al., 1995; Alvarez et al., 2002; Huggett et al., 2004; Lambert et al., 2007, 2010). Alterations in norepinephrine transport and central sympathetic outflow have also been implicated in postural tachycardia syndrome (Lambert et al., 2008), anxiety and cardiovascular disease (Esler et al., 2006b), as well as panic disorder (Lambert et al., 2002). Furthermore, studies have shown that epi-/peri-cardial adipose tissue is an independent risk factor for AF (Al Chekakie et al., 2010) and adipocytes from the epicardial fat directly infiltrate into the myocardial wall resulting in enlargement of the ventricular myocardium and atrial septum thereby increasing the risk of AF (Iacobellis et al., 2003; Sarin et al., 2008; Mahabadi et al., 2009). Another highly relevant link in the current context is the noradrenaline-mediated increase in sodium glucose co-transporter- 2 (SGLT-2) expression (Matthews et al., 2017). This is important given that SGLT-2 inhibitors such as canagliflozin effectively reduce epicardial fat (Yagi et al., 2017) and empagliflozin improved glucose control and has been demonstrated to reduce cardiovascular events in T2DM (Zinman et al., 2015).

Sympathetic blockade at the periphery can also be achieved by beta-adrenergic blockers. Though beta-blockers are not generally regarded as membrane stabilizing agents, they delay atrial repolarization and protect against AF. It was speculated that beta-blockers suppress the pulmonary vein ectopy that triggers AF (Chen et al., 1999) as well as protect against adrenergically mediated shortening of the action potential duration (APD) which precipitates and maintains AF (Kühlkamp et al., 2000). Beta-blockers are a good first choice for the control of the ventricular response in AF and are widely used in the prevention of AF in patients following cardiothoracic surgery, in which AF occurs in approximately 30% of patients (Prystowsky et al., 1996).

### Device-Based Therapeutic Approaches for Sympathetic Inhibition

Other interventions include device-based therapeutic approaches to modulate ANS to lower central sympathetic nerve activity such as renal denervation (RDN) and carotid body ablation. These approaches are primarily aimed at reducing sympathetic activity and BP in resistant hypertensive patients but are now investigated as potential treatment strategies in the context of AF.

## Renal Denervation and Ganglionated Plexi Ablation

Recently, the AFFORD study, a single-arm pilot study including 20 patients with symptomatic paroxysmal or persistent AF, suggested that RDN alone was safe and able to decrease AF burden in min/day as measured using an implantable cardiac monitor (ICM) at 12-month follow-up accompanied by an improvement in quality of life (Feyz et al., 2018). A clinical study that assessed the antiarrhythmic effect of RDN in addition to pulmonary vein isolation (PVI) in HTN patients with symptomatic AF demonstrated that a mean BP reduction of 5–10 mmHg led to a 7% decrease in AF burden as measured with an ICM (Romanov et al., 2017). Pokushalov et al. (2012) showed that RDN on top of PVI in patients with symptomatic AF and resistant HTN reduced the incidence of AF recurrence significantly. At 1 year 69% of the patients in the PVI + RDN group were free of AF episodes, while in the PVI only group, only 29% of the patients remained free of AF episodes (Pokushalov et al., 2012).

Mechanistic studies demonstrated that modulation of the SNS via RDN improved AF control (Hou et al., 2013). Linz et al. (2013a,b) demonstrated the effect of RDN on heart rate (RR-interval, 708 ± 12 ms (∼85 bpm) vs. 577 ± 19 ms (∼104 bpm), post- and pre-procedure respectively; *P* = 0.0021) and ventricular rate response in pigs with permanent AF. The authors described a reduction of 24% in ventricular rate response in the treated pigs vs. sham, as well shorter AF episodes post-RDN compared with sham (12 ± 3 vs. 34 ± 4 s; *P* = 0.0091) (Linz et al., 2013a,b). Effects of RDN on cardiac electrophysiology have already been extensively studied and found to be effective in animal models and humans (Okin et al., 2006; Symplicity HTN-2 Investigators et al., 2010; Brandt et al., 2012b). RDN predominantly impacts sympathetic tone and results in a reduction in heart rate and atrioventricular-conduction velocity in resistant hypertension population (Krum et al., 2009; Schlaich et al., 2010; Ukena et al., 2013). Recently, the SPYRAL HTN-OFF MED trial reported that the higher baseline heart rate was associated with higher BP decrease, which may indicate that RDN works better in patients with high sympathetic drive (assessed in the absence of interfering drugs) (Bohm et al., 2018).

In a prospective, longitudinal study of patients with controlled hypertension, paroxysmal AF and either normal renal function or chronic kidney disease (CKD), Kiuchi et al., applying an extensive ablation technique reported the incidence of AF recurrence. It was higher in CKD patients treated with PVI alone (61.5%) than in CKD patients treated with PVI + RDN (38.5%, *P* = 0.0251) or non- CKD patients who underwent PVI (35.6%, *P* < 0.0001) over 22.4 ± 12.1 months following intervention. In addition, they found an improvement in echocardiographic parameters such as indexed left atrial volume, left ventricular end-diastolic diameter, and left ventricular mass index in patients who underwent RDN compared to baseline and to other groups. Another important finding was that patients with CKD stage 4 who underwent only PVI (64.41%) demonstrated a higher rate of AF recurrence than those subjected to PVI + RDN (33.33%, *P* = 0.0409) (Kiuchi et al., 2017).

Chronic kidney disease and AF share risk factors and putative mechanisms suggesting that common pathophysiologic processes may drive both pathologies. One possible common link between AF and CKD is activation of the renin–angiotensin–aldosterone system (RAAS) (Goette et al., 2000; Tsai et al., 2004; Wachtell et al., 2005; Baber et al., 2011; Tsai et al., 2017). Angiotensin II can increase atrial pressure, promote atrial fibrosis, and modulate ion channels, all of which are involved in structural and electrical remodeling of the atria resulting in AF (Goette et al., 2000). In addition, gene polymorphisms in encoding components of this pathway have been linked to the development of AF (Tsai et al., 2004; Lubitz et al., 2014). Supporting these results are data demonstrating that BP reduction after RDN is associated with improvements in regional and global atrial conduction with reduction in ventricular mass and fibrosis (McLellan et al., 2015). Though the antiarrhythmic effects of RDN could be due to a synergistic effect of better BP control, withdrawing this important risk factor for AF recurrence, improves AF control by electrophysiological modification such as the prolongation of the atrial effective refractory period (Manolis et al., 2012; Wang et al., 2015).

**FIGURE 3:**
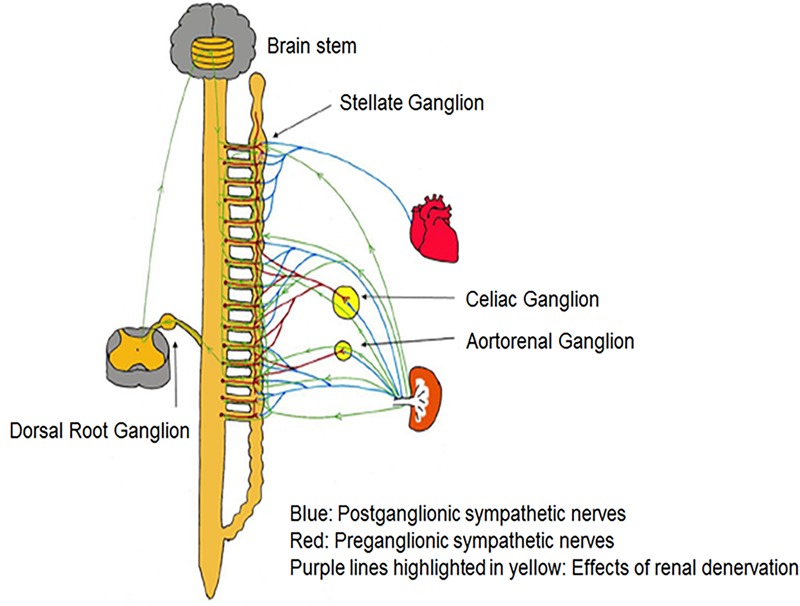
There are multiple pathways connecting renal sympathetic nerves with the stellate ganglion. Both preganglionic and postganglionic sympathetic fibers may innervate the renal artery.

Tsai et al. (2017) demonstrated that bilateral RDN, possibly via interrupting afferent renal innervation, led to substantial brain stem and bilateral stellate ganglion remodeling at 8 weeks’ post-procedure in ambulatory canines (Tsai et al., 2017). These changes were associated with reduced ^18^FDG (fluorodeoxyglucose) – uptake in the brainstem, left stellate ganglion nerve activity and atrial tachyarrhythmic events. It was proposed that neural remodeling in the brain stem and stellate ganglion may partially explain the described antiarrhythmic effects of RDN (Tsai et al., 2017). *Trans*-synaptic degeneration is a phenomenon in the central and peripheral nervous system that may remain active both at the level of the insult and in remote brain structures for as long as 1 year following trauma (Bramlett and Dietrich, 2007) resulting in long-term functional consequences following RDN as shown in Figure 3. Meckler and Weaver (1984) showed that approximately 10% of bilateral renal sympathetic neurons in cats originated from the thoracic chain ganglia (stellate through T13). Because of the connections between these two structures, RDN may directly result in retrograde cell death of the stellate ganglion. Furthermore, the application of fluorescent dyes in the renal nerves results in fluorescent labeling of the sympathetic cell bodies in paravertebral and prevertebral ganglia (Ferguson et al., 1986; Gattone et al., 1986; Sripairojthikoon and Wyss, 1987).

Since the sympathetic preganglionic neurons that project to the stellate ganglion are dispersed in spinal cord segments sections T1–T10 (Pilowsky et al., 1992), they have ample chances to interrelate with the preganglionic cells that link indirectly with sympathetic nerve fibers surrounding the renal arteries. However, some other pathways might contribute to the *trans*-synaptic degeneration (Tsai et al., 2017), as the ganglion cells of renal afferent nerves in thoracic and lumbar spine dorsal root ganglia link to the posterior and lateral hypothalamic nuclei as well as the locus ceruleus in the brain stem (Campese and Kogosov, 1995; Jansen et al., 1995). RDN may affect the described connections and result in remodeling of the critical brainstem areas and the stellate ganglia. Because transneuronal degeneration may remain active for prolonged periods of time, the effects of RDN on arrhythmia control may persist for months after the procedure (Tsai et al., 2017).

## PV Isolation

Pulmonary vein isolation with ganglionated plexi ablation has been used in autonomic modulation of interactions between the systemic and intrinsic cardiac ANS. PVI combined with autonomic ganglia modification significantly improved success rates after PVI compared to PVI alone (Katritsis et al., 2011). In animal models of vagal induced AF, the effectiveness of pulmonary vein isolation was due to the ablation of the autonomic ganglia at the base of the pulmonary veins which eliminated rapid pulmonary vein firing in response to high-frequency stimulation of the ganglionated plexi (Lu et al., 2009). GP ablation inhibited AF inducibility in sleep apnea animal models (Ghias et al., 2009) and has been used in patients with both paroxysmal and persistent AF alone or combined with pulmonary vein isolation with better success rate in patients with paroxysmal AF (Pokushalov et al., 2009; Katritsis et al., 2011; Mikhaylov et al., 2011). However, though ganglionated plexi ablation appears to be a safe and efficacious adjunctive technique to improve outcomes of pulmonary vein isolation in patients with paroxysmal AF, GP ablation in animal models after acute myocardial ischemia exhibited ventricular arrhythmogenic effects compared with GP ablation of the normal heart (He et al., 2013). Hence further studies are warranted to investigate the application of these procedures in various cardiac conditions.

## Botulinum Toxin Injection

Recent studies have reported that an abundance of epicardial fat is associated with direct adipocyte infiltration into the underlying atrial myocardium (Hatem and Sanders, 2014; Mahajan et al., 2015). Moreover, increased overall adiposity has been shown to be associated with shortened effective refractory periods in the pulmonary veins, leading to the speculation that adiposity can predispose to AF initiation (Munger et al., 2012). Indeed, there is some evidence that areas of epicardial fat accumulation correlate with sites of high dominant frequency, suggesting that epicardial fat may influence AF triggers (Nagashima et al., 2012; Nakahara et al., 2014). Also, it may be possible that encasing epicardial fat influences ganglionated plexi and thus contributes to arrhythmogenesis. Recently, in a prospective, double-blind randomized controlled clinical trial (Pokushalov et al., 2015) reported that injections of botulinum toxin into epicardial fat pads in patients undergoing coronary artery bypass grafting (CABG) resulted in a substantial reduction in the incidence of atrial tachyarrhythmia (most pronounced in the first 14 months postoperatively) and AF burden throughout the 3-year follow-up period, accompanied by reduction in need for hospitalization (Pokushalov et al., 2015; Romanov et al., 2018).

## Stellate Ganglion Blockade

Studies demonstrate that stimulation of the stellate ganglion increased sinus rate and predisposes to atrial arrhythmias (Mohan et al., 2001; Tan et al., 2008a,b). Increased extrinsic cardiac nerve activity in the left stellate ganglion often preceded paroxysmal atrial tachycardia or AF in animal models (Choi et al., 2010). Unilateral electrical stimulation of the stellate ganglion aggravated atrial electrical remodeling facilitating induction of AF in animal models, an effect attenuated by unilateral ganglionectomy (Zhou et al., 2013). In animal models of pacing-induced congestive heart failure, cryoablation of bilateral stellate and T2–T4 thoracic ganglia reduced sympathetic activation-induced paroxysmal AF (Ogawa et al., 2009). Bilateral ANS remodeling resulting from the increased synaptic density of stellate ganglia following myocardial infarction was associated with increased ganglion activity (Han et al., 2012). Surgical excision of stellate ganglion (left cardiac sympathetic denervation) together with T2 and T3 thoracic ganglia reduced arrhythmia in high-risk patients and animal models following myocardial infarction (Schwartz et al., 1976, 1992) and in patients with catecholaminergic polymorphic ventricular tachycardia and long-QT syndrome (Collura et al., 2009).

## High Thoracic Epidural Anesthesia

In patients undergoing cardiac surgery and cardiopulmonary bypass, high thoracic epidural anesthesia was found to markedly reduce sympathetic tone (Scott et al., 2001; Liu et al., 2004) and the risk of post-operative supraventricular arrhythmias (Svircevic et al., 2011). Initiation of high thoracic epidural anesthesia decreased the arrhythmia burden in patients with refractory electrical storm (Bourke et al., 2010) and in animal models of rapid atrial pacing was found to be associated with inhibition of atrial autonomic nerve sprouting (Yang et al., 2011). However variable results have been obtained pertaining to the decreased incidence of post-operative sustained AF despite a marked reduction in sympathetic activity (Groban et al., 2000; Jidéus et al., 2001) and warrants further detailed studies to explore the definitive effect of high thoracic epidural anesthesia on atrial electrophysiology and arrhythmogenesis.

## Carotid Body Ablation

Hypersensitive chemoreceptors result in sympathetic excitation. Enhanced chemoreflex sensitivity potentially increasing central sympathetic drive was observed in animal models of pacing-induced congestive heart failure (Schultz and Li, 2007). Surgical denervation of peripheral chemoreceptors prevented sympathetic activation- mediated hypertensive response to hypoxic stimuli in rats with intermittent hypoxia (Lesske et al., 1997). Bilateral denervation of the carotid body and the carotid sinus baroreceptors prevented the development of hypertension in young pre-hypertensive animals and significantly decreased arterial pressure in the adult population of spontaneously hypertensive rats (Tan et al., 2010) and hypertensive humans (Nakayama, 1961). Though carotid ablation appears as a logical approach to attenuate sympathetic overdrive, hypertension and arrhythmogenic atrial autonomic signaling in AF, there are currently no studies characterizing its effects on atrial electrophysiology and atrial arrhythmogenesis.

## Conclusion and Perspectives

Enhanced sympathetic activation increases circulating catecholamines and causes hypertension and associated complication such as AF and congestive heart failure. Hypertension often coexists with comorbidities like obesity, metabolic syndrome, and obstructive sleep apnea that further exaggerate and sustain the sympathetic overdrive. Substantial evidence indicates that targeting sympathetic overactivity either by pharmacotherapy or device-based interventions seems to be a logical and useful approach for the management of AF. Sympathetic ablation by RDN appears to be a promising strategy to achieve atrial antiarrhythmic effects in animal models and humans. While carotid body ablation is a proven strategy to attenuate sympathetic overdrive, the potential anti-arrhythmic effects are yet to be investigated in future studies. Other techniques employed to reduce sympathetic activity are applied in specific conditions such as stellate blockade in high-risk patients and following myocardial infarction, ganglionic plexus ablation to lower AF recurrence in patients following circumferential pulmonary vein isolation, and high thoracic epidural anesthesia in post-surgical AF management. However further clinical studies are needed to substantiate the role of various sympatholytic approaches in the management of AF.

## Author Contributions

RC and MS have drafted the manuscript. MK, JH, and VM have researched data, contributed to inidvidual sections, and critically reviewed the manuscript.

## Conflict of Interest Statement

MS is supported by an NHMRC Research Fellowship and has received consulting fees, and/or travel and research support from Medtronic, Abbott, Novartis, Servier, Pfizer, and Boehringer-Ingelheim. The remaining authors declare that the research was conducted in the absence of any commercial or financial relationships that could be construed as a potential conflict of interest.
